# Drug-resistant cell lines in cancer

**DOI:** 10.1038/s41467-024-52441-z

**Published:** 2024-09-25

**Authors:** Jindrich Cinatl, Mark N. Wass, Martin Michaelis

**Affiliations:** 1 Dr Petra Joh Research Institute, Komturstraße 3 A, 60528 Frankfurt am Main, Germany; 2 School of Biosciences, University of Kent, Canterbury, CT2 7NJ UK

**Keywords:** Cancer therapy, Cancer models

## Abstract

This poster demonstrates the development of drug-resistant cancer cell lines and their application in cancer research.

Over the years, clinical landscape of patients with cancer has dramatically changed due to the development of new and refinement of existing therapies. However, while patients may initially respond favorably to treatment, the development of resistance remains a major limitation. To understand and identify approaches to overcome mechanism of resistance, the availability of suitable resistant cancer models is critical. This post from Nature Communications illustrates how drug-resistant cancer cell lines can be developed and discusses how they can be used to address the issue of cancer therapy resistance.
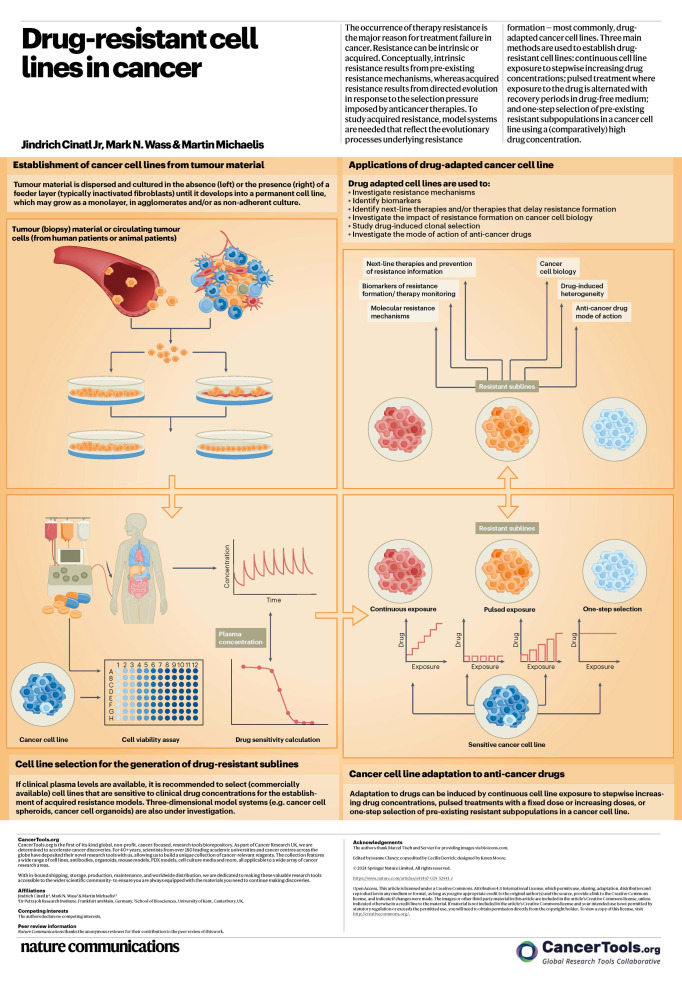


This poster is freely available online thanks to support from CancerTools.org.

The poster has been peer reviewed and Springer Nature retains sole responsibility for all editorial content.

